# Change in Swallowing Function and Substance P Levels Associated with Nicergoline in Neurological Disease: A Pilot Study

**DOI:** 10.3390/jcm15124728

**Published:** 2026-06-18

**Authors:** Jutikan Imsub, Pasiri Sithinamsuwan, Chanasak Hathaiareerugand, Yarnisar Sakunchit, Juthathip Suphanklang

**Affiliations:** 1 The College of Pharmacotherapy of Thailand, Nonthaburi 11000, Thailand; goku2744@gmail.com; 2 Department of Pharmacy Practice and Pharmaceutical Care, Faculty of Pharmaceutical Science, Burapha University, Chonburi 20131, Thailand; 3 Division of Neurology, Department of Medicine, Phramongkutklao Hospital, Bangkok 10400, Thailand; pasiripmk@gmail.com (P.S.); bobby_med@hotmail.com (Y.S.); 4 Division of Physical Medicine & Rehabilitation, Department of Medicine, Phramongkutklao Hospital, Bangkok 10400, Thailand; chanasak.h@pcm.ac.th; 5 Division of Pharmaceutical Care, Silpakorn University, Nakhon Pathom 73000, Thailand

**Keywords:** dysphagia, ischemic stroke, nicergoline, substance P, IPW, GUSS

## Abstract

**Background/Objectives**: Dysphagia is a prevalent consequence of neurological conditions, particularly stroke and Parkinson’s disease, leading to aspiration pneumonia and reduced quality of life. Currently, there are no specific recommendations for pharmacological therapy, although studies indicate that elevated substance P levels may improve swallowing function. While nicergoline is known to increase substance P, the role of its major metabolite, 10-methoxy-dihydro-lysergol (MDL), in this therapeutic effect remains unclear. This study examined the therapeutic effects of nicergoline and its correlation with substance P and MDL levels. **Methods**: This study conducted an open-label pilot study with historical controls in neurogenic patients with dysphagia. The primary outcome was improvement in the Gugging Swallowing Screen (GUSS) scores at week 12. Secondary outcomes included choking frequency, serum substance P levels, and the correlation between serum MDL levels and dysphagia enhancement. Inverse Probability Weighting (IPW) was employed to adjust for baseline confounders. **Results**: A total of 92 patients were analyzed: 26 in the nicergoline group (20 or 60 mg/day) and 66 in the historical control group. Compared to controls, the nicergoline group exhibited significantly higher median of GUSS scores (20 (IQR: 19–20) vs. 15 (IQR: 9–19), *p* < 0.001) and significantly lower median of choking frequency (6.43 (IQR 0–17) vs. 108 (IQR 13–201) 105.22, *p* < 0.001). The median substance P concentration in the therapy group was 4089.15 (IQR: 3336.13–4468.26) pg/mL. Patients receiving nicergoline showed a statistically significant elevation in substance P from baseline (*p* < 0.001). Pearson analysis revealed a negligible correlation between serum MDL and substance P levels (R^2^ = 0.0349). **Conclusions**: Preliminary findings suggest that nicergoline may be associated with improvements in swallowing function in neurogenic dysphagia and a potential increase in substance P levels. The lack of correlation with serum MDL suggests that efficacy may not linearly depend on circulating metabolite concentrations. Further large-scale randomized controlled trials are warranted.

## 1. Introduction

Dysphagia is a frequent and often severe complication that can be life-threatening, particularly among the elderly population. Commonly reported adverse outcomes associated with dysphagia include aspiration pneumonia, malnutrition, and prolonged hospitalization. While dysphagia can arise from various etiologies, neurogenic dysphagia swallowing impairment resulting from neurological dysfunction represents a significant etiology. The swallowing process is a highly complex neurological mechanism, requiring coordinated neural activity across multiple levels of the central nervous system, extending from the cerebral cortex to the brainstem [1,2]. The epidemiological data highlight the high prevalence of dysphagia among patients with neurological disorders. Specifically, the prevalence of dysphagia in stroke patients is approximately 65%, followed by Parkinson’s disease (PD) at approximately 50%. These two conditions represent the highest occurrences of swallowing dysfunction [3].

Swallowing has oral, pharyngeal, and esophageal stages. Due to oral and pharyngeal abnormalities, stroke patients often develop dysphagia. Modern clinical data suggests both cortical and brainstem infarctions may produce dysphagia, even though the brainstem is the main swallowing area. Recent investigations have shown that periventricular white matter abnormalities induce dysphagia. Lesions may affect anterior–posterior neuronal signaling. Abnormalities in gray matter intraneuronal communication may damage integrated neural networks essential for coordinated swallowing [4,5].

Gray matter lesions, especially basal ganglia infarction, may produce dysphagia. This disorder is caused by dopaminergic and substance P pathway dysfunction. Reduced dopamine metabolism alters swallowing and cough reflexes by disrupting substance P-mediated afferent signaling pathways within vagal sensory neurons, rather than merely depressing local peptide concentrations [6,7]. The main swallowing brain circuits are nigrostriatal, striatonigral, and striatopallidal. Enkephalin controls the striatopallidal pathway, whereas substance P and dynorphin control nigrostriatal fibers signaling. Swallowing is substantially affected by substantia nigra afferent transmission. Subthalamic nucleus stimulates substantia nigra, limiting swallowing. Striatonigral substantia nigra suppression aids swallowing [4].

PD causes basal ganglia degeneration and a substantia nigra dopamine decrease. Nearly all Parkinson’s patients have dysphagia. Recent investigations have compared Parkinson’s disease and cerebrovascular disease blood substance P levels. Parkinson’s disease patients exhibited significantly lower serum substance P levels than the cerebrovascular group. The substantia nigra’s diminished dopaminergic output may have down-regulated substance P expression [8].

Angiotensin-converting enzyme inhibitors (ACEIs) helped stroke patients to swallow. ACEIs increase peripheral neuron substance P concentration by suppressing Angiotensin-converting enzyme (ACE). Baseline substance P deficit following partial basal ganglia injury may benefit from this medication [8]. Parkinson’s patients may swallow better with levodopa. Increased substance P levels from substantia nigra dopaminergic activation enhance therapeutic outcomes. Modern therapies increase substance P using ACEIs, TRPV1 agonists, and Levodopa [2].

Mechanistically, nicergoline exhibits a strong binding affinity to α1-adrenoceptors and serotonin 5-HT1A receptors (IC50 = of 0.2 and 6 nmol/L, respectively), alongside a moderate affinity for α2-adrenoceptors and serotonin 5-HT2 receptors [9,10]. Through these receptor interactions, nicergoline enhances the release and turnover of noradrenaline and dopamine across various brain regions [9,11]. Crucially, clinical evidence by Nishiyama and Nakashima has demonstrated that oral nicergoline administration significantly elevates serum substance P levels, thereby suggesting a therapeutic role in improving the swallowing reflex and reducing neurogenic dysphagia [12,13].

Following oral ingestion, nicergoline undergoes rapid and extensive first-pass metabolism, demethylating from 1-methyl-10-alpha-methoxy-9,10-dihydrolysergol (MMDL) to its major active metabolite, 1,6-dimethyl-8β-hydroxymethyl-10α-ergoline (MDL) [10]. Given that MDL accounts for approximately 51% of the parent compound and possesses the longest elimination half-life of 11 to 20 h [10], this study specifically focused on measuring its concentrations alongside substance P to further explore their clinical relationship.

Nicergoline’s primary metabolites, MDL and MMDL, have unknown pharmacological effects and receptor affinity. D1 and D2 receptors may bind MDL and MMDL, although the evidence is inconsistent. Moretti et al. found that nicergoline and MMDL bind to dopamine receptors, whereas MDL mainly affects noradrenergic activity [14,15]. This may explain the metabolite’s dopaminergic affinity.

This study is the first-ever comparison of substance P levels and dysphagia treatment efficacy between nicergoline and control groups. The reliable and straightforward Gugging Swallowing Screen (GUSS) assessed swallowing [16]. Compared to Fiberoptic Endoscopic Evaluation of Swallowing, GUSS [16], nearly 100% sensitivity was observed in neurological patients [16,17,18,19]. In addition to GUSS [16] tests at weeks 1, 4, and 12, patients or caregivers reported daily choking. This longitudinal data confirmed swallowing progress or stability despite clinical evaluation complicating factors including patient anxiety.

## 2. Materials and Methods

### 2.1. Participants and Eligibility

Participants were recruited from the Neurological Outpatient Department at Phramongkutklao Hospital from January 2023 until February 2025. After obtaining informed consent, a total of 92 patients with neurogenic dysphagia were enrolled in this study.

To be eligible for inclusion, participants were required to meet the following criteria: age over 20 years, a confirmed diagnosis of either ischemic stroke with an onset of ≥2 months or Parkinson’s disease, no recent usage of nicergoline and a commitment to maintaining stable baseline medications. Conversely, patients were excluded from the study if they presented with chronic dyspepsia, gout, acute gouty attack, renal impairment, hepatic dysfunction, hypotension, bradycardia, active bleeding, or advanced malignancy. Additionally, individuals with a history of laryngopharyngeal, gastrointestinal, or central nervous system surgery, as well as those on prolonged bedrest, were excluded. Patients who demonstrated non-compliance with ACEIs or dopaminergic medications for less than 2 months were also excluded from the analysis.

Following enrollment, all patients underwent baseline and follow-up GUSS examinations. Furthermore, daily choking diaries were maintained by the participants to facilitate longitudinal clinical assessment.

### 2.2. Study Design

This was a pilot study with historical controls involving neurogenic patients with dysphagia, and open-label research. Participants were allocated into two groups at a 1:2.5 ratio: the nicergoline group (*n* = 26, administered 20 or 60 mg/day) and the control group (*n* = 66). Clinical assessments were performed at weeks 4 and 12 over the 12-week follow-up period. In the nicergoline group, a pharmacist provided the prescription and monitored for side effects, while medication adherence was confirmed by pill counts at each visit. At baseline, all subjects received GUSS [16] evaluations and blood collection to determine baseline serum substance P levels. At weeks 4 and 12, both groups were re-evaluated using the GUSS [16] and choking diaries to determine swallowing effectiveness. In the nicergoline group, serum substance P levels were measured and compared to baseline at week 4. Blood samples for the analysis of MDL concentrations were collected at two time points to characterize the pharmacokinetic profile including the trough concentration (Ctrough) or concentration at 12 h post-dose and the peak concentration (Cpeak) or concentration at 3–4 h post-dose in all participants. In addition, some participants voluntarily consented to provide blood samples during the elimination phase to measure drug concentrations at 5–7 h (Celimination 5–7) and 8–11 h (Celimination 8–11) post-dose.

### 2.3. Measurement Outcome

The primary outcome was to assess the GUSS [16] scores between the nicergoline group and the control group at the 12-week follow-up. The secondary outcomes included a comparative examination of the incidence of choking events recorded in choking diaries across the two groups, an evaluation of serum substance P levels and their correlation with the improvement in dysphagia in the nicergoline cohort at the 4-week follow-up relative to baseline, and an exploration of serum MDL concentrations (Cpeak and Ctrough) and the improvement in dysphagia in the nicergoline group at the 4-week follow-up. An experienced evaluator used the GUSS [16] to determine dysphagia severity. Scores range from 0 to 20, with four severity levels: no dysphagia (20 points, normal diet, minimal aspiration risk), slight (15–19 points, semisolid and liquid intake allowed), moderate (10–14 points, semisolid diet only), and severe (0–9 points, nothing per oral (NPO) advised). This research designated the transition to a group with superior scoring as ‘dysphagia improvement’.

Blood samples for serum substance P and MDL concentrations were kept in Ethylenediaminetetraacetic acid (EDTA) and citrate tubes. The samples were centrifuged and stored at −80 °C until analysis. ELISA (Abcam, Cambridge, UK) measured serum substance P levels [20], whereas liquid chromatography–mass spectrometry (LC-MS) measured MDL values [21,22]. Plasma concentrations of MDL were assessed utilizing liquid chromatography–mass spectrometry with electrospray ionization (LC/MS-ESI). The analyte was identified and monitored through its unique mass spectrum at a mass-to-charge ratio (*m*/*z*) of 255 [21] (see Figure S2 in the Supplementary Materials). The resultant spectral peak intensities were translated into plasma concentrations employing a linear regression equation obtained from a 7-point calibration curve [22].

This study followed the GCP criteria and was approved by the Phramongkutklao Hospital Institutional Review Board. Everyone gave written informed permission before enrolling. Before recruiting the first participant, ClinicalTrials.gov registered the experiment (NCT05551182) on 22 September 2022. This study reports an exploratory pilot analysis of the registered trial (NCT05551182). Due to the limited sample size (*n* = 26), all participants receiving nicergoline were analyzed as a pooled treatment group to maintain adequate statistical power.

### 2.4. Statistical Analysis

As this was an exploratory pilot study, a strict formal sample size calculation was not performed to power the primary hypothesis. The sample size of 92 patients was determined based on feasibility and clinical availability during the study period, aiming to provide baseline variance and effect-size data for future definitive large-scale trials.

To address the potential selection bias and residual confounding arising from the non-randomized allocation, IPW was applied to the analysis of GUSS scores and choking frequencies across all study visits, including baseline. The propensity scores used to generate the weights were derived from a logistic regression model.

Descriptive statistics were utilized to summarize the data; categorical variables were expressed as frequencies and percentages, whereas continuous variables were presented as medians and interquartile ranges (IQRs). Inter-group comparisons for non-parametric continuous data were performed using the Mann–Whitney U test, and the chi-squared test (or Fisher’s exact test, where appropriate) was employed for categorical variables. Statistical significance was defined as a *p*-value < 0.05. All statistical analyses were executed using IBM SPSS Statistics for macOS, version 29.0 (IBM Corp., Armonk, NY, USA).

## 3. Results

### 3.1. Study Populations

The baseline characteristics of 92 research subjects are reported in Table 1. Most metrics were similar between nicergoline and control groups before statistical adjustment. Regarding potential concurrent medication baseline characteristics, angiotensin-converting enzyme inhibitors (ACEIs) usage was minimal and did not significantly differ between the two groups (15.38% in the nicergoline group vs. 6.06% in the historical control group; *p* = 0.231.

**Figure 1:**
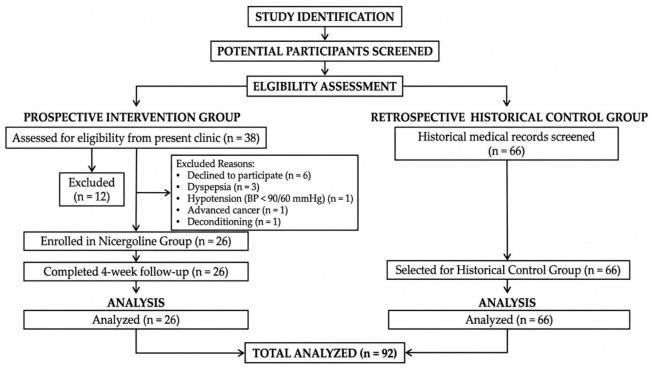
Participant flowchart.

The nicergoline group had significantly higher baseline GUSS scores than the control group (median: 16 (IQR: 14–16) vs. 9 (IQR: 9–11); *p* < 0.001). Choking was significantly decreased in the nicergoline group (median: 148 (IQR: 144–156) vs. 219 (IQR: 152–243); *p* < 0.001). IPW corrected these baseline imbalances. Subsequent to IPW modification, the two groups attained statistical equivalence. GUSS scores after correction were 11 (IQR: 9–15) for nicergoline and 10 (IQR: 9–16) for control. (*p* = 0.768). The adjusted choking-frequency median for the nicergoline group was 211 (IQR: 148–231) and that of the control group was 188 (IQR: 121–234) (*p* = 0.124). Only the nicergoline group had baseline blood substance P levels measured before medication, with a median value of 2222.50 pg/mL. A participant flow chart is presented in Figure 1.

Prior to IPW, the standardized mean difference (SMD) for the baseline GUSS score was 2.05, indicating a substantial imbalance between the nicergoline and control groups. After applying IPW, the SMD was drastically reduced to 0.04, which is well below the conventional threshold of 0.1, demonstrating that the IPW model effectively balanced this baseline covariate. Similarly, the baseline SMD for choking frequency was 0.76 before weighting, indicating a significant initial disparity. The application of IPW reduced the SMD for this variable to 0.31. While this represents a notable reduction in the initial imbalance, the post-weighting SMD remained above the 0.1 threshold.

Before weighting, GUSS stage distribution differed significantly between groups (*p* < 0.001), with substantial imbalances in Stage 1 (SMD = 1.26), Stage 2 (SMD = 0.64), and Stage 3 (SMD = 1.56. After IPW adjustment, the distribution became balanced *p* = 0.310). The post-weighting SMDs for Stage 1 and Stage 3 fell below the 0.1 threshold (0.09 and 0.07, respectively), indicating an effective balance, while Stage 2 was partially reduced to 0.28.

The propensity scores were calculated using a logistic regression model including the following covariates: GUSS score, stage of GUSS score and choking frequency. To prevent influence from extreme weights, stabilized weights (or truncated weights at the 1st and 99th percentiles) were applied. The effective sample size after weighting was calculated to be 164.

### 3.2. Impact of Nicergoline on Neurogenic Dysphagia

Statistical correction using the IPW approach was conducted to provide a fair comparison between the nicergoline and control groups (see Table S1 in the Supplementary Materials). The assessment of nicergoline’s therapeutic effect on dysphagia, as measured by GUSS scores, revealed that the nicergoline group exhibited a significantly higher median GUSS score compared to the control group (20 (IQR: 19–20) vs. 15 (IQR: 9–19), *p* < 0.001). The nicergoline group had a markedly lower median for choking than the control group (6.43 (IQR 0–17) vs. 108 (IQR 13–201), *p* < 0.001). Consequently, the nicergoline group demonstrated higher GUSS scores and fewer choking incidents compared to the control group (Table 2). Nicergoline treatment was associated with a significantly greater clinical improvement in dysphagia symptoms compared to the natural recovery observed in the historical control group.

### 3.3. The Correlation Between GUSS, Serum Substance P and Serum MDL in Nicergoline Group

Post-treatment serum substance P levels at week 4 were available for 18 out of the 26 patients in the nicergoline group. The remaining eight samples were missing due to insufficient serum sample volume to perform the ELISA assay. A sensitivity analysis confirmed that baseline clinical characteristics did not differ significantly between patients with available data and those with missing samples, indicating a minimal risk of attrition bias.

**Figure 2:**
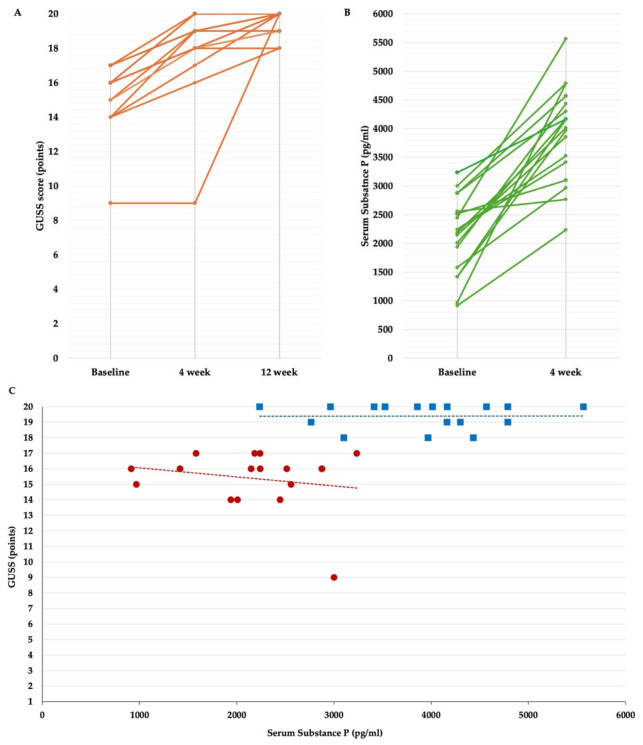
Individual patient analysis of GUSS and serum substance P level changes. (**A**) Changes in individual GUSS scores from baseline to 12 weeks following nicergoline administration in patients with neurogenic dysphagia. (**B**) Changes in individual serum substance P from baseline to 4 weeks following nicergoline administration in patients with neurogenic dysphagia. (**C**) Individual changes in GUSS scores and serum substance P levels. Significant increases were observed 4 weeks after nicergoline administration (blue squares) compared to baseline (orange circles).

Figure 2B, the median substance P level was 4089.15 pg/mL (range: 3442.24–4401.64), representing a median increase of 1876.88 pg/mL (IQR: 1770.93–1853.64 pg/mL) from baseline. Eighteen patients in the nicergoline group showed a substantial increase in blood substance P levels (t = −8.40, *p* < 0.001, 95% CI: 1343.82–2237.51). GUSS scores improved significantly with nicergoline (Figure 2A). At 12 weeks, the median GUSS score was 20 (range 19–20), up 1.5 points (range 1–2). All eighteen patients (100%) showed improved GUSS. No patient lost swallowing function. The statistical analysis showed a substantial improvement (t = −9.68, *p* < 0.001, 95% CI: 2.04–3.18). Cohen’s d = 1.28 showed a significant clinical effect. Pearson’s correlation was not statistically significant (r = 0.11, *p* = 0.66), but the data’s unidirectionality was. Without exception, all patients’ Substance P and GUSS scores increased simultaneously, confirming the clinical concept that Substance P aids in swallowing recovery (Figure 2C).

**Figure 3:**
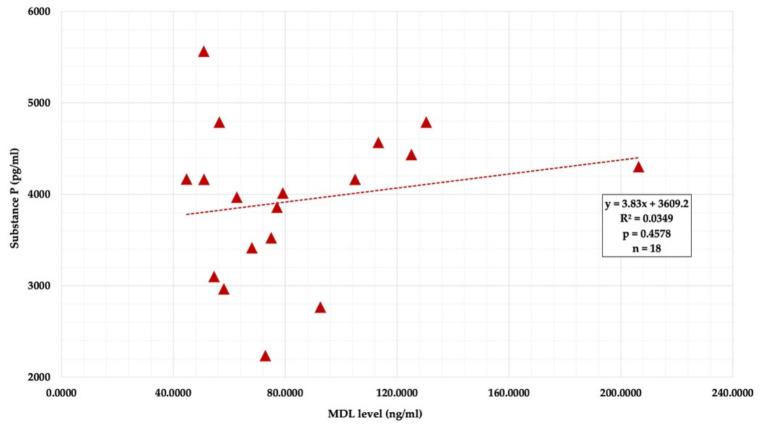
The correlation between serum MDL (Cpeak) and serum substance P levels after nicergoline intake.

The calibration curve showed remarkable linearity, validated by a correlation coefficient (r^2^) of 0.9995, thereby ensuring elevated analytical precision and reliability throughout the investigated range (see Figure S1 in the Supplementary Materials). The plasma concentrations of MDL following oral administration of nicergoline at doses of 20 mg and 60 mg are summarized in Table 3. The median (interquartile range, IQR) values for Cpeak, Ctrough, and Celimination were 73.92 (56.82–101.81) ng/mL, 34.48 (23.45–44.69) ng/mL and 49.84 (42.85–60.55) ng/mL, respectively (see Table S2 in the Supplementary Materials). In addition, the relationship between serum MDL level and serum substance P level was not statistically significant, and the confidence interval suggested the true effect could range from negative to positive (95% CI include zero). The extremely low R^2^ (0.0349) indicated that there was no linear correlation between serum MDL and substance P levels. This finding supports our hypothesis that the parent drug (nicergoline), rather than its MDL metabolite, is the primary driver of substance P elevation (Figure 3).

### 3.4. Sensitivity Analysis for Clinical Heterogeneity

To evaluate the potential impact of clinical heterogeneity introduced by the inclusion of patients with PD, a dedicated sensitivity analysis was performed by restricting the IPW analysis exclusively to the post-stroke subgroup (completely excluding all patients with PD).

The between-group comparison demonstrated that the primary therapeutic outcomes remained exceptionally robust and unaltered. Following the exclusion of the Parkinson’s cohort, patients in the nicergoline group exhibited highly statistically significant improvements compared to the control group. Specifically, post-treatment GUSS scores at 12 weeks were significantly higher in the nicergoline group (*p* < 0.001), and the choking frequency over the 12-week observation period was drastically and significantly lower in the nicergoline group compared to the controls (*p* < 0.001). Furthermore, within the nicergoline group, plasma substance P concentrations exhibited a highly significant post-treatment elevation (*p* = 0.002) following the exclusion of the Parkinson’s cohort. These definitive findings confirm that the clinical efficacy of nicergoline on neurogenic dysphagia is consistently sustained and independently significant within the post-stroke population (Table 2).

## 4. Discussion

This study’s results show that nicergoline helps stroke patients with dysphagia get better. The GUSS scores of patients in the nicergoline group showed a large increase from their starting levels. There was a statistically significant difference in GUSS scores between the nicergoline group and the control group. These results are consistent with earlier studies, including the research conducted by Nakashima et al., which utilized the 2-step water-swallowing Test (2ST) to evaluate dysphagia recovery [13]. Their study also showed that nicergoline made the 2ST results better. The 2ST is only good for measuring how much water someone drinks, and it cannot tell if someone is silently aspirating. On the other hand, the GUSS advises a more thorough evaluation by looking at three consistencies: semi-solid, liquid, and solid. By starting the test with semi-solids, the GUSS instrument makes it easier to find several indicators of silent aspiration, like delayed swallowing, drooling, and changes in voice [16,26]. Daily choking diaries and GUSS evaluations made dysphagia tests better. GUSS offers a standardized, clinician-rated evaluation of swallowing function, whereas choking diaries record real-time symptoms, necessitating this dual methodology. Cross-validation of formal clinical assessments and longitudinal patient-reported outcomes fortified this study’s findings.

Additionally, biochemical examination of the nicergoline group revealed a statistically significant elevation in serum substance P levels at 4 weeks post-intervention compared to baseline. This biochemical boost was supported by a contemporaneous and considerable reduction in clinical dysphagia symptoms, demonstrated by elevated GUSS scores and a decrease in choking occurrences. These results are consistent with prior studies that showed nicergoline medication increases serum Substance P levels and improves swallowing abilities, as evidenced by the 2ST [12,13].

To ascertain the biological mechanism, nicergoline may increase substance P levels by promoting dopaminergic turnover, potentially improving swallowing neural circuits [2,8]. The current investigation demonstrated a greater elevation in substance P compared to two previous assessments. The disparity may be elucidated by the baseline substance P levels and the severity of dysphagia among the study subjects. Previous research focused on persons with moderate-to-severe dysphagia [12], evaluated using the 2-step swallowing test, although this study demonstrated mild-to-moderate dysphagia, indicated by a mean GUSS score of 16. Patients exhibiting decreased dysphagia may possess augmented dopaminergic–neuropeptide pathways, potentially elevating baseline substance P levels after nicergoline therapy [2,8]. Stroke-related and Parkinson’s disease-related dysphagia possess distinct pathophysiological mechanisms. Nevertheless, the sensitivity analysis restricted to the post-stroke subgroup demonstrated that nicergoline administration was significantly associated with dysphagia improvement. Furthermore, this sub-analysis confirmed that, within the nicergoline group, plasma substance P concentrations exhibited a highly significant post-treatment elevation, even after the exclusion of PD patients.

This study revealed no significant association between blood concentrations of MDL, the primary metabolite of nicergoline, and serum substance P levels at 4 weeks. In addition, pharmacokinetic parameters (Cpeak, Ctrough, Celimination) exhibited no correlation with substance P elevation, and elevated MDL concentrations did not consistently correspond with increasing substance P levels [14,15]. Some patients in the low-dose group had higher substance P levels than those in the high-dose group. Although MDL is recognized as the primary long-acting metabolite of nicergoline, its direct correlation with the biomolecular shifts in this study warrants a deeper look into the parent compound itself. Data suggest that nicergoline, the parent compound, may regulate substance P overexpression via dopaminergic processes, rather than MDL. Pharmacological evidence indicates that MDL primarily influences noradrenergic pathways, while MMDL exhibits a greater affinity for dopaminergic receptors; nonetheless, both metabolites are less powerful than the parent compound [15]. Previous studies have demonstrated that nicergoline is extensively and rapidly converted into its active metabolites, MDL and MMDL [21,22]. As a result, the parent chemical is often undetectable in systemic circulation when assessed using conventional liquid chromatography–electrospray ionization mass spectrometry (LC/MS-ESI) methods [21]. Because of the lack of ability to directly quantify the parent drug, this study’s findings are dependent on MDL concentrations [22]. The absence of a dose–response correlation with MDL tentatively suggests that the parent drug, nicergoline, likely predominantly influences substance P overexpression through dopaminergic mechanisms [15]. However, since parent nicergoline concentrations were not directly quantified in the current protocol, this mechanistic interpretation remains strictly hypothesis-generating and must be interpreted with caution. In vitro results further support that metabolic conversion substantially reduces the overall pharmacological efficacy of nicergoline [14,21,22]. Future investigations must refine blood-sampling procedures to precisely determine the pharmacokinetic relationship between nicergoline and MDL. The fast metabolism of the parent chemical needs earlier initiation of blood collection and increased sampling frequency. Moreover, the concurrent measurement of both nicergoline and MDL utilizing extremely sensitive analytical methodologies, such as liquid chromatography–tandem mass spectrometry (LC-MS/MS) [27,28], would be necessary to definitively validate these tentative conclusions and establish a precise pharmacokinetic–pharmacodynamic (PK-PD) relationship.

Several limitations should be considered when interpreting the findings of this study. First, although patients using prescribed substance P-modulating medications (such as ACEIs) were maintained on stable regimens, the use of over-the-counter preparations, including topical capsaicin or dextromethorphan, was not fully documented. These unaccounted medications could have introduced variability into the observed substance P levels.

Second, the study utilized a hybrid design combining a prospective nicergoline cohort with a retrospective historical control group. Although IPW was rigorously applied to mitigate selection bias and balance baseline characteristics, this non-randomized approach cannot entirely eliminate residual confounding. Specifically, prior to weighting, substantial imbalances existed for the baseline GUSS score (unweighted SMD = 2.05), choking frequency (unweighted SMD = 0.76), and GUSS severity stages (Stage 1 SMD = 1.26; Stage 2 SMD = 0.64; Stage 3 SMD = 1.56). While the application of IPW successfully minimized disparities for the overall GUSS score (post-weighting SMD = 0.04), GUSS Stage 1 (post-weighting SMD = 0.09), and GUSS Stage 3 (post-weighting SMD = 0.07) to well below the conventional 0.1 threshold, the post-weighting SMDs for choking frequency and GUSS Stage 2 remained elevated, at 0.31 and 0.28, respectively. These residual imbalances indicate that the IPW model could not fully eliminate disparities across all baseline variables, presenting potential sources of residual confounding. As a pilot and exploratory study, these methodological constraints mean the findings should be interpreted with caution.

Third, plasma substance P concentrations were exclusively monitored within the nicergoline group, as the historical control cohort lacked available biorepository samples for biochemical tracking. Consequently, the observed longitudinal increase in substance P cannot be definitively isolated from potential confounding influences, such as natural neurological recovery, time-dependent physiological changes, or the synergistic effects of background medications. Although GUSS and choking frequency were strictly compared between groups using an IPW-weighted model, the single-arm nature of our biomarker analysis means these biochemical findings should be considered exploratory.

Regarding potential concurrent medication confounders, ACEIs are known to upregulate substance P and potentially improve swallowing reflexes. Because ACEI exposure was low and distributed without statistically significant imbalance, its potential to act as a major confounder is substantially minimized. This finding strengthens the hypothesis that the significant elevation of substance P and the corresponding functional improvements observed in the treatment cohort are primarily associated with nicergoline administration rather than background ACEI therapy.

Finally, we acknowledge the inherent clinical heterogeneity within our sample due to the inclusion of both post-stroke and PD patients under the umbrella of neurogenic dysphagia, alongside a relatively small sample size typical of an exploratory pilot study, which might restrict the broader generalizability of our conclusions. Although these conditions differ in their underlying neurological pathways and the restricted sample size poses constraints, our exploratory sensitivity analysis focused strictly on the post-stroke subgroup demonstrated highly consistent and statistically significant improvements across all primary biochemical and clinical endpoints. This robust alignment between clinical swallowing outcomes and biochemical markers strongly supports the validity of these preliminary findings.

Future investigations should employ fully prospective, randomized controlled designs with larger sample sizes and standardized medication monitoring to definitively validate these therapeutic effects.

GUSS scores, incidents of choking, and serum substance P concentrations all showed improvement with nicergoline administration. Standardized swallowing evaluations, continuous symptom monitoring, and IPW-adjusted variable analysis need further refinement in future studies. The gap between MDL exposure and substance P response enhances pharmacological comprehension by demonstrating that the parent drug, rather than its metabolites, exerts the strongest therapeutic effect. This study associated functional recovery to neurochemical modulation with favorable nicergoline as an adjuvant for neurogenic dysphagia; nevertheless, larger prospective cohorts are necessary.

In conclusion, the findings from this preliminary study suggest that nicergoline therapy is associated with improvements in swallowing function among patients with neurogenic dysphagia, potentially accompanied by an increase in serum Substance P levels. These observations tentatively point toward a dopaminergic or neuromodulatory pathway. Given that MDL exposure did not demonstrate a clear correlation with Substance P changes, the direct involvement of the parent compound remains an open hypothesis. Consequently, considering the exploratory, open-label design of this pilot investigation, further robust, prospective, and randomized controlled trials are essential to definitively validate these tentative conclusions.

## Figures and Tables

**Table 1:** Baseline characteristics.

	Nicergoline (N = 26)	Control (N = 66)	*p*-Value	SMD
Age, mean (SD)	72.73 (11.09)	73.76 (13.10)	0.287	
Male, *n* (%)	16 (61.53)	44 (66.67)	0.637	
Stroke, *n* (%)	22 (84.60)	59 (89.39)	0.498	
Stroke localization				
Supratentorial, *n* (%)	21 (80.80)	59 (89.39)	0.309	
Brainstem, *n* (%)	2 (7.70)	1 (1.51)	0.192	
NIHSS, median (IQR)	8 (5–12)	7 (5–11)	0.827	
Duration of stroke (months), median (IQR)	6.50 (4.00–9.50)	5.00 (4.00–11.00)	0.720	
Parkinson’s disease, *n* (%)	7 (26.90)	12 (31.82)	0.396	
Hoehn and Yahr *			0.326	
2, *n* (%)	2 (7.70)	6 (9.10)		
3, *n* (%)	4 (15.40)	6 (9.10)		
4, *n* (%)	1 (3.80)	0 (0.00)		
Stroke with Parkinson’s disease, *n* (%)	3 (11.54)	5 (7.58)	0.683	
GUSS (unweighted), median (IQR)	16 (14–16)	9 (9–11)	<0.001	2.05
GUSS (weighted), median (IQR)	11 (9–15)	10 (9–16)	0.768	0.04
GUSS stage (unweighted)			<0.001	
1, *n* (%)	1 (3.80)	34 (51.52)		1.26
2, *n* (%)	6 (23.10)	24 (36.36)		0.64
3, *n* (%)	19 (73.10)	8 (12.12)		1.56
GUSS stage (weighted)			0.310	
1, *n* (%)	23 (30.30)	37 (34.40)		0.09
2, *n* (%)	26 (26.3)	28 (36.8)		0.28
3, *n* (%)	36 (36.4)	25 (32.9)		0.07
Choking frequency (total episode over 4 weeks) ** (unweighted), median (IQR)	148 (144–156)	219 (152–243)	<0.001	0.76
Choking frequency (total episode over 4 weeks) ** (weighted), median (IQR)	211 (148–231)	188 (121–234)	0.124	0.31
Substance P (pg/mL) ***, median (IQR)	2222.50 (1540.50–2637.86)	Not available		
ACEIs, *n* (%)	4 (15.38)	4 (6.06)	0.231	

Abbreviations: ACEIs, Angiotensin-Converting Enzyme Inhibitors; GUSS [16], Gugging Swallowing Screen; IQR, interquartile range; n/a, not applicable; NIHSS [23,24], National Institutes of Health Stroke Scale; SD, standard deviation. * Hoehn and Yahr [25], a clinical staging scale for assessing the severity of Parkinson’s disease symptoms. ** Choking frequency represents the cumulative number of choking episodes recorded by patients in their daily diaries over the 4-week follow-up period. *** Substance P levels were not available for the historical control group.

**Table 2:** Comparative efficacy of nicergoline in the treatment of dysphagia: IPW-adjusted analysis of GUSS scores and daily choking frequency.

	Group	Weighted			Unweighted		
		N	Median (IQR)	*p*-Value	N	Median (IQR)	*p*-Value
Main Analysis: Overall Cohort (Neurogenic Dysphagia Includes Post-Stroke and PD)							
GUSS (primary outcome)	Nicergoline	75	20.00 (19.00–20.00)	<0.001	26	20 (19–20)	<0.001
	Control	89	15.00 (9.00–19.00)		66	13.00 (9.00–17.00)	
Choking (total episode over 12 weeks)	Nicergoline	75	6.43.00 (0.00–17.00)	<0.001	26	13.00 (2.00–21.00)	<0.001
	Control	89	108.00 (13.00–201.00)		66	110.00 (17.00–217.00)	
Substance P level (pg/mL)	Nicergoline	-	-		26	4089.15 (3336.13–4468.26)	<0.001 *
Sensitivity Analysis: Post-Stroke Subgroup (Excludes Parkinson’s Disease)							
GUSS (primary outcome)	Nicergoline	66	19.00 (19.00–20.00)	<0.001	19	19 (19.0–20.0)	<0.001
	Control	82	15.60 (10.00–19.00)		54	13.50 (9.0–17.0)	
Choking (total episode over 12 weeks)	Nicergoline	66	87.00 (9.09–181.78)	<0.001	19	13.00 (2.00–17.00)	<0.001
	Control	82	3.00 (0.00–15.00)		54	87.00 (10.50–215.5)	
Substance P level (pg/mL)	Nicergoline	-	-		19	4164.55 (3525.28–4733.95)	0.002 *

Abbreviation: GUSS [16], Gugging Swallowing Screen; * Within-group pre- and post-treatment comparison for the nicergoline arm analyzed via the Related-Samples Wilcoxon Signed-Rank Test.

**Table 3:** Plasma concentration of MDL at peak, trough and elimination concentration in nicergoline group.

Plasma MDL Concentration	Range of Plasma Level
Cpeak, ng/mL	44.72–206.30
Ctrough, ng/mL	6.74–63.73
Celimination, ng/mL	31.34–84.62

Abbreviations: Cpeak: the peak concentration of MDL; Ctrough: the trough concentration of MDL; Celimination: the elimination concentration of MDL; ng/mL: nanograms per milliliter.

## Data Availability

The datasets used and/or analyzed during the current study are available from the corresponding author on reasonable request.

## Supplementary Materials

The following supporting information can be downloaded at: https://www.mdpi.com/article/10.3390/jcm15124728/s1, Table S1: Balance diagnostics of covariates before and after Inverse Probability of Treatment Weighting (IPW); Table S2: Balance diagnostics of covariates before and after Inverse Probability of Treatment Weighting (IPW); Figure S1: the calibration curve of plasma MDL concentration. The graph illustrates the linear relationship between MDL concentration (ng/mL) and the area under the curve (AUC) obtained from LC/MS-ESI analysis. The 7-point calibration curve shows high linearity with a regression equation y = 4827.5x + 82,705 and an R2 value of 0.9995; Figure S2: The mass spectra of standard MDL.

## Abbreviations

The following abbreviations are used in this manuscript:

2-ST	2-step water-swallowing
5-HT1A	Serotonin 1A
5-HT2	Serotonin 2
95% CI	95% confidence interval
ACE	Angiotensin-converting enzyme
ACEIs	Angiotensin-converting enzyme inhibitors
Celimination	Elimination concentration
Cpeak	Peak concentration
Ctrough	Trough concentration
D1	Dopamine 1
D2	Dopamine 2
ELISA	Enzyme-linked immunosorbent assay
GCP	Good clinical practice
GUSS	Gugging swallowing screen
IC50	Half-maximal inhibitory concentration
IPW	Inverse probability weighting
IQR	Interquartile
LC-MS	Liquid chromatography–mass spectrometry
LC/MS-ESI	Liquid chromatography–mass spectrometry with electrospray ionization
LC-MS/MS	Liquid chromatography–tandem mass spectrometry
M1	Muscarinic 1
M2	Muscarinic 2
MDL	10-methoxy-dihydro-lysergol
MMDL	10-alpha-meth from 1-methyl-10-alpha-methoxy-9,10-dihydrolysergol
*m*/*z*	Mass-to-charge ratio
NIHSS	National Institutes of Health Stroke Scale
ng/mL	Nanogram per milliliter
PD	Parkinson’s disease
pg/mL	Picogram per milliliter
PK–PD	Pharmacokinetic–pharmacodynamic
SD	Standard deviation
SMD	Standardized mean difference
TRPV1	Transient Receptor Potential Vanilloid 1
