# Supplementary material for: Change in Swallowing Function and Substance P Levels Associated with Nicergoline in Neurological Disease: A Pilot Study
Source: J Clin Med. 2026 Jun 18;15(12):4728. doi: 10.3390/jcm15124728 (PMC13301109; doi:10.3390/jcm15124728)
Supplement: Supplementary file 1 [file jcm-15-04728-s001.zip › jcm-4305437-supplementary.pdf]

Table S1 Balance diagnostics of GUSS score and choking frequency (total episode over 4 weeks) before and after Inverse Probability of Treatment Weighting (IPW)

	**Group**	**N (unweighted)**	**Pseudo N (weighted)**	**SMD (unweighted)**	**SMD (IPW)**
**GUSS**	Nicergoline	26	75		
	Control	67	89	2.05	0.04
**Choking** frequency (total episode over 4 weeks)	Nicergoline	26	75		
	Control	67	89	0.76	0.31

Prior to IPW, the standardized mean difference (SMD) for the GUSS was 2.05, that mean substantial imbalance between the Nicergoline and control groups. After IPW, the SMD was reduced to 0.04, which is well below the conventional threshold of 0.1, show that the IPW model effectively balanced the GUSS covariate.

Similarly, the SMD for the Choking was 0.76 before weighting, indicating a significant baseline difference. The application of IPW reduced the SMD for this variable to 0.31. While this represents a reduction in the imbalance, the post-weighting SMD remained above the 0.1 threshold, suggesting that the IPW model was less effective at fully balancing the Choking covariate.

Table S2 Serum levels of MDL (Cpeak) and substance P in neurogenic dysphagia patients

	N	Mean ± SD	Median (IQR)	Range
**Serum MDL (Cpeak) level** (ng/mL)	18	84.60 ± 39.98	73.92 (56.66-103.60)	44.72 – 206.30
**Serum substance P level** (pg/mL)	18	3,933.01 ± 819.07	4,089.15 (3,402.58-4,375.63)	2,236.00 – 5,565.95

The data showed high inter-individual variability in both serum MDL (Cpeak) level (CV = 47.2%) and serum substance P levels (CV = 20.8%). The distribution of serum MDL level showed slight positive skewness with a wide range (44.72 - 206.30 ng/mL), indicating substantial pharmacokinetic variability among patients. The 95% confidence interval for the slope includes zero, confirming that there is no significant linear relationship between serum MDL level and serum substance P levels.

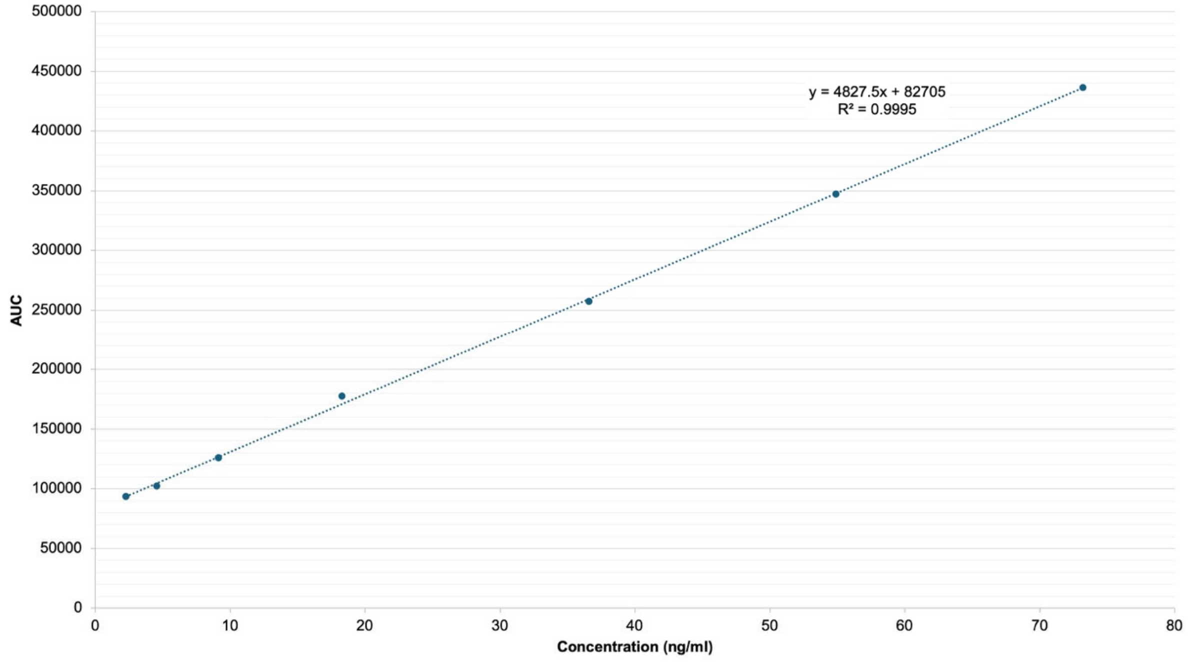

Figure S1 The calibration curve of plasma MDL concentration. The graph illustrates the linear relationship between MDL concentration (ng/mL) and the area under the curve (AUC) obtained from LC/MS-ESI analysis. The 7-point calibration curve shows high linearity with a regression equation y = 4827.5x + 82705 and an R^2 value of 0.9995.

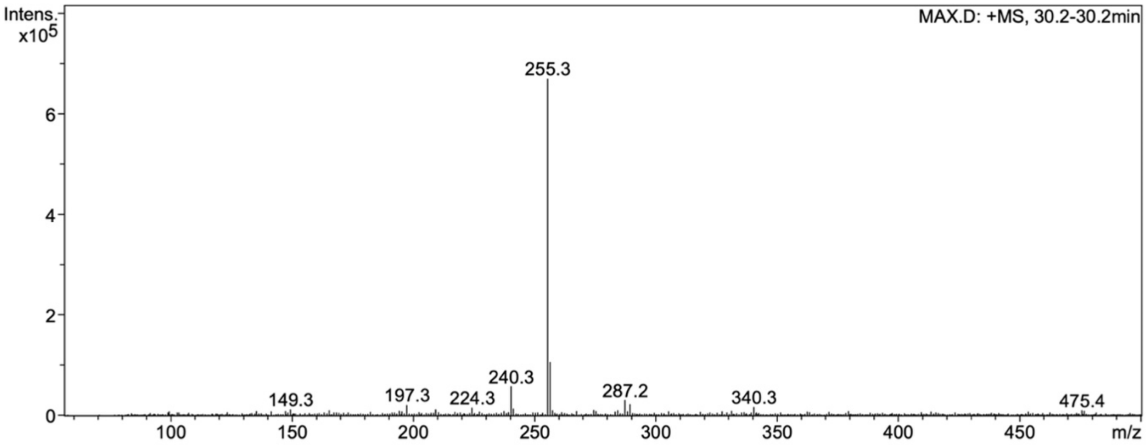

Figure S2 The mass spectra of standard MDL
